# Optimization of Protein Extraction from Sunflower Meal Using Taguchi Design and Regression Modeling for Human Nutrition Applications

**DOI:** 10.3390/foods14142415

**Published:** 2025-07-08

**Authors:** Anca Becze, Marin Senila, Lacrimioara Senila, Lucian Dordai, Oana Cadar, Vanda Liliana Fuss-Babalau, Marius Roman, Levente Levei, Paul Uiuiu, Mihai Octavian Naghiu

**Affiliations:** 1 National Institute for Research and Development of Optoelectronics INOE 2000, Research Institute for Analytical Instrumentation, 67 Donath Street, RO-400293 Cluj-Napoca, Romania; marin.senila@icia.ro (M.S.); lacri.senila@icia.ro (L.S.); oana.cadar@icia.ro (O.C.); vanda.fuss@icia.ro (V.L.F.-B.); marius.roman@icia.ro (M.R.); levente.levei@icia.ro (L.L.); 2 Department of Fundamental Sciences, University of Agricultural Sciences and Veterinary Medicine Cluj-Napoca, 3–5 Mănăştur Street, RO-400372 Cluj-Napoca, Romania; paul.uiuiu@usamvcluj.ro; 3 Department of Teacher Training, Technical University of Cluj-Napoca, 15 Constantin Daicoviciu Street, RO-400020 Cluj Napoca, Romania; naghiumihai@gmail.com

**Keywords:** sunflower meal, protein extraction, response surface methodology, alternative proteins, functional food ingredient, amino acid profile, optimization, sustainable nutrition

## Abstract

In response to the growing demand for sustainable protein sources, this study explores the valorization of sunflower meal—a by-product of oil extraction—as a protein-rich ingredient suitable for human nutrition. The aim was to optimize the extraction process and assess the nutritional and safety profile of the resulting protein flour. Mechanical stirring, ultrasound-assisted, and CO_2_-assisted extraction methods were evaluated, with mechanical stirring selected for optimization due to its scalability and energy efficiency. A Taguchi L9 orthogonal array was employed to evaluate the effects of pH, temperature, and sample mass on protein content. A first-order regression model was developed and validated (R^2^ = 0.86, *p* < 0.05), identifying optimal conditions at pH 10.0, 30 °C, and 60 g per 500 mL of distilled water. Under these conditions, protein content reached 49.87%. The extracted protein flour exhibited improved nutritional quality with high protein content, moderate solubility (53.4%), and favorable amino acid composition—particularly rich in glutamic acid, aspartic acid, and arginine. Safety analyses confirmed the absence of detectable aflatoxins and very low PAH levels. These results support the use of sunflower protein concentrate as a sustainable, nutritionally valuable, and safe ingredient for functional food applications. Further studies are recommended to improve functional properties and assess sensory acceptance.

## 1. Introduction

The growing global population, combined with increasing environmental concerns, has intensified the need for sustainable protein sources to meet future nutritional demands [1,2]. Conventional animal-based proteins, including meat and dairy products, are associated with significant environmental impacts, such as high greenhouse gas emissions, intensive land and water use, and biodiversity loss [3,4]. As a result, there is an urgent need to diversify protein sources beyond traditional pathways [5].

Alternative proteins derived from plants, microorganisms, and agro-industrial by-products offer promising opportunities to develop sustainable and nutritionally adequate food systems [6,7]. Their production typically requires fewer natural resources and generates a lower environmental footprint compared to animal-based proteins, aligning with global sustainability goals [8].

In this context, international initiatives such as the European Green Deal advocate for a transition toward more sustainable agricultural and food production systems [9]. Valorizing agro-industrial by-products such as sunflower meal supports circular economy principles and sustainable protein supply for human nutrition [10,11,12].

Sunflower meal, a by-product generated after oil extraction from sunflower seeds (*Helianthus annuus* L.), represents one of the most abundant agro-industrial residues produced worldwide [13]. Rich in proteins, fibers, and minerals, sunflower meal is currently used predominantly as a component in animal feed formulations [14,15]. However, its potential for human nutrition remains largely untapped [16]. With up to 50% protein, sunflower meal offers a promising plant-based protein source for diet diversification [17]. Furthermore, sunflower proteins exhibit a favorable amino acid profile, with relatively high levels of essential amino acids such as methionine and cysteine compared to other oilseed meals [18]. Despite these advantages, the direct incorporation of sunflower meal into human foods is limited by factors such as high fiber content, presence of antinutritional factors, and variability in functional properties [19,20]. Specifically, sunflower meal proteins often exhibit low solubility and poor emulsifying capacity, which restrict their application in many food formulations. These functional limitations reduce their effectiveness in products such as beverages, sauces, or emulsified meat analogs. Therefore, optimizing extraction conditions is critical not only for improving protein yield and purity but also for enhancing the techno-functional properties of the recovered proteins, such as solubility and emulsification potential, which are essential for broadening their use in human nutrition. Efficient extraction and purification strategies are thus essential to recover high-quality proteins suitable for food applications [21]. Valorizing sunflower meal for human consumption aligns with the principles of resource efficiency and sustainable food production [22]. It offers an opportunity to enhance the nutritional value of food products while promoting circular economy practices by reducing waste and maximizing the use of agricultural resources [23].

The recovery of high-quality proteins from sunflower meal for human nutrition applications presents several technological and functional challenges [24]. The complex matrix of sunflower meal, characterized by a high fiber content and the presence of phenolic compounds, can hinder protein solubility, extraction efficiency, and downstream functionality [25]. Moreover, conventional extraction processes may result in low yields, partial denaturation of proteins, or co-extraction of unwanted compounds that negatively affect the nutritional and sensory properties of the final products. Optimizing the extraction conditions—including critical parameters such as pH, temperature, and solid-to-liquid ratio—is essential to maximize protein content while preserving the structural integrity and functional properties of the proteins. Tailoring these parameters enables the development of efficient, scalable, and sustainable extraction processes, unlocking the full nutritional potential of sunflower proteins for incorporation into high-value food applications. The establishment of optimized extraction protocols is therefore a key step toward enhancing the valorization of sunflower meal within the framework of sustainable food systems.

The application of statistical and mathematical modeling tools, such as Response Surface Methodology (RSM), provides a powerful approach for optimizing extraction processes. RSM enables the systematic evaluation of multiple process variables and their interactions, facilitating the identification of optimal operating conditions with a reduced number of experimental trials. By constructing predictive models and response surfaces, it becomes possible to visualize and interpret the complex relationships between input factors, such as pH, temperature, and solid-to-liquid ratio, and key response variables like protein content and quality. This methodology not only improves the efficiency and precision of process development but also supports the design of robust, reproducible, and scalable extraction protocols. In the context of protein recovery from sunflower meal, the use of modeling and optimization techniques contributes to the advancement of sustainable food processing technologies and the effective valorization of agro-industrial by-products.

The specific objective of this study was to optimize the alkaline extraction of proteins from sunflower meal by evaluating the effects of pH, temperature, and sample mass on protein yield. To achieve this, we employed a Taguchi L9 orthogonal array, which offers a statistically robust and resource-efficient design for identifying the most influential process parameters with a limited number of experimental trials. This approach was selected over more complex techniques such as Response Surface Methodology (RSM) due to its suitability for preliminary optimization and emphasis on main effects. The ultimate goal was to establish optimal processing conditions that enhance protein recovery while preserving nutritional value, improving functional attributes, and ensuring food safety. The resulting sunflower protein concentrat was comprehensively characterized in terms of proximate composition, amino acid profile, solubility, mineral content, and contaminant levels to evaluate its potential application as a safe, functional, and sustainable ingredient in human nutrition.

## 2. Materials and Methods

### 2.1. Samples Collection

The sunflower meal used in this study was sourced from five different commercial oil processing facilities located in Romania. The samples were collected to reflect a representative range of processing conditions and raw material quality. All samples were stored in sealed containers at room temperature prior to analysis. For each experimental condition, tests were performed in triplicate using pooled material prepared by homogenizing equal portions from each of the five sources. This approach ensured both experimental reproducibility and broader applicability of the results.

### 2.2. Reagents and Materials

All solvents used were of HPLC grade and purchased from VWR (Darmstadt, Germany). Ultra-pure water was obtained using the ULTRACLEAR UV UF EVOQUA purification system (Pittsburgh, PA, USA). The ACW Kit for antioxidant capacity determination was sourced from Analytik Jena (Jena, Germany). Immunoaffinity columns for aflatoxin analysis (3 mL AFLASTAR^®^ IA) and the BIOPURE Mycotoxin Mix 1 (Aflatoxins) in acetonitrile (5 mL) were obtained from Romer Labs (Butzbach, Germany).

The OPA reagent kit, containing 10 mg/mL each of o-phthalaldehyde and 3-mercaptopropionic acid in 0.4 M borate buffer (6 × 1 mL), was supplied by Agilent Technologies (Santa Clara, CA, USA). Additional reagents included sodium hydroxide CS reagent (≥97.0%, pellets), Folin–Ciocalteu’s phenol reagent, PAH Calibration Mix certified reference material (10 μg/mL each component in acetonitrile), gallic acid monohydrate (ACS reagent, ≥98.0%), L-ascorbic acid BioXtra (≥99.0%, crystalline), and the Amino Acid Standard, all purchased from Sigma-Aldrich (Saint Louis, MO, USA).

### 2.3. Protein Extraction

Protein extraction from sunflower meal was performed using three distinct methods: mechanical stirring, ultrasound-assisted extraction (UAE), and CO_2_-assisted extraction. Each method was selected to optimize protein recovery while minimizing the environmental impact of the process.

For comparative purposes, a fixed amount of 50 g of sunflower meal was mixed with 500 mL of distilled water, and the pH was adjusted to 10.5 using a 10 percent NaOH solution. This fixed ratio was used for all three extraction methods to allow baseline comparison prior to the optimization phase described in Section 3.3, where sample mass, pH, and temperature were systematically varied. In addition, this standard condition, based on values commonly reported in the literature, served as a control reference to evaluate the performance of the optimized extraction parameters. The following extraction conditions were applied:

#### 2.3.1. Mechanical Stirring

Extraction was carried out for 20 min at 400 rpm using a mechanical stirrer (OS20-Pro, DLAB, Johor, Malaysia) while maintaining the temperature at 40 °C with a magnetic stirrer hot plate (RET basic, IKA, Staufen, Germany).

#### 2.3.2. Ultrasound-Assisted Extraction

The extraction was performed in an ultrasonic bath (SONOREX, Bandelin, RK 103H, Berlin, Germany) operating at 59 kHz for 20 min, with the temperature controlled at 40 °C.

#### 2.3.3. CO_2_-Assisted Extraction

High-pressure CO_2_ extraction was conducted using a 1-L benchtop reactor equipped with a 4875 Power Controller (Parr Instrument Company, Moline, IL, USA). The extraction was performed for 20 min at a pressure between 55.5 and 57.8 bar and a temperature between 20.7 and 22.3 °C, corresponding to subcritical CO_2_ conditions. Subcritical CO_2_ was chosen due to its ability to act as a gentle, non-toxic, and residue-free extraction medium that promotes the release of intracellular compounds by penetrating the matrix and disrupting cell structures without the use of organic solvents. Although CO_2_ does not directly solubilize proteins, its physical interaction with the plant matrix—especially under pressure and agitation—facilitates protein release by enhancing mass transfer and cell wall permeability. An agitation speed of 11,000 rpm was used to intensify this effect. Following extraction, the liquid phase was separated from the solid residue by decantation. To facilitate protein precipitation, ascorbic acid was gradually added to the liquid extract to adjust the pH to between 3.5 and 4.0. The resulting mixture was then centrifuged at 5000 rpm for 10 min (Megafuge 16, Thermo Fisher Scientific, Waltham, MA, USA) to separate the precipitated proteins. The protein precipitate was collected and subsequently lyophilized using a freeze-dryer (FreeZone 6 Liter −50 C, Labconco Corporation, Kansas City, MO, USA) to obtain a stable powdered product. The lyophilized proteins were stored at 4 °C in airtight containers until further analysis.

### 2.4. Ash Content Analysis

Ash content was determined according to the ISO 2171:2023 standard [26]. Approximately 5 g of the homogenized sample was accurately weighed into a pre-dried crucible and incinerated in a muffle furnace at 550 °C until complete combustion was achieved. After cooling the crucible in a desiccator to room temperature, the residue was weighed. Ash content was calculated gravimetrically and expressed as a percentage of the initial sample weight.

### 2.5. Moisture Content Analysis

Moisture content was determined according to the ISO 11294:1994 standard [27]. Approximately 5 g of sample was dried at 103 °C in a UFE 400 laboratory oven (Memmert, Büchenbach, Germany) until a constant weight was achieved. Moisture content was calculated gravimetrically and expressed as a percentage of the initial sample weight.

### 2.6. Total Lipid Content (Soxhlet Extraction Method)

The total lipid content was determined using a simplified Soxhlet extraction method. Approximately 5 g of dried and homogenized sample was placed in an extraction thimble and extracted with petroleum ether in a Soxhlet apparatus for 4–6 h. Following extraction, the solvent was evaporated, and the lipid residue was dried at 105 °C to constant weight. The lipid content was calculated gravimetrically and expressed as a percentage of the initial sample weight.

### 2.7. Total Protein Content (Kjeldahl Method)

The total protein content (TPC) was analyzed according to the ISO 937:2023 standard [28]. 1 g of homogenized sample was digested with 15 mL of concentrated sulfuric acid and a catalyst mixture in a Kjeldahl digestion unit at 350–400 °C until a clear solution was obtained. After digestion, the sample was neutralized with 40% sodium hydroxide solution and distilled using a Kjeldahl distillation unit. Ammonia was collected in a 4% boric acid solution and titrated with 0.1 N hydrochloric acid using bromocresol green/methyl red indicators. The nitrogen content was determined and converted to protein content using a conversion factor of 6.25, which is commonly applied for plant-based proteins and assumes an average nitrogen content of 16% in protein molecules. This approach is in accordance with ISO 937:2023 and AOAC 981.10 guidelines, and results were expressed as a percentage of the initial sample weight [28,29].

### 2.8. Total Dietary Fiber Determination

Total dietary fiber content was determined according to AOAC Method 985.29 [30]. A quantity of 1.0 g of homogenized sample was weighed into a 400 mL beaker and mixed with 40 mL of MES-TRIS buffer solution, composed of 0.05 M 2-(N-morpholino)ethanesulfonic acid and 0.05 M Tris(hydroxymethyl)aminomethane, adjusted to pH 8.3. The sample was subjected to enzymatic digestion by sequential addition of 50 μL of thermostable α-amylase (≥300 U/mL), followed by incubation in a boiling water bath (100 °C) for 30 min with occasional stirring. After cooling to 60 °C, 100 μL of protease solution (≥50 U/mL) was added, and the mixture was incubated for another 30 min. Finally, 5 mL of amyloglucosidase solution (≥300 U/mL) was added, and incubation continued at 60 °C for 30 min. Following enzymatic hydrolysis, 95% ethanol preheated to 60 °C was added in a volume four times greater than the digest to precipitate the soluble fibers. The mixture was allowed to stand at room temperature for 60 min before being filtered through a pre-weighed glass fiber filter (0.5 μm pore size). The residue was washed sequentially with two 15 mL portions of 78% ethanol, two 15 mL portions of 95% ethanol, and two 15 mL portions of acetone. The filter and residue were then dried at 105 °C to constant weight, cooled in a desiccator, and weighed. Total dietary fiber content was calculated gravimetrically and expressed as a percentage of the initial sample weight. Blank corrections were applied by processing a parallel sample without any test material.

### 2.9. Polycyclic Aromatic Hydrocarbon (PAH) Analysis

The determination of polycyclic aromatic hydrocarbons (PAHs) was performed using an adapted method based on the procedure described by Nieva-Cano et al., 2001 [31]. High-performance liquid chromatography (HPLC) with fluorimetric detection (FLD) was employed following sonication-assisted extraction.

Analysis was conducted using a Perkin Elmer 200 Series HPLC system (Waltham, MA, USA) equipped with a fluorescence detector. Separation of 15 PAHs—including naphthalene, acenaphthene, fluorene, phenanthrene, anthracene, fluoranthene, pyrene, benzo(a)anthracene, chrysene, benzo(b)fluoranthene, benzo(k)fluoranthene, benzo(a)pyrene, dibenzo(a,h)anthracene, benzo(g,h,i)perylene, and indeno(1,2,3-cd)pyrene—was achieved on an Inertsil ODS-P column (5 μm, 4.6 × 150 mm, GL Sciences, Tokyo, Japan) maintained at 24 °C.

The mobile phase consisted of a gradient system of ultrapure water and acetonitrile. The injection volume was 50 μL. A time-programmed fluorescence detection method was applied to selectively detect different PAHs. Results were expressed as nanograms per gram (µg/kg) of sample.

### 2.10. Aflatoxin Analysis

Aflatoxin content was determined according to EN ISO 16050:2011 [32]. Briefly, a 25 g sample was extracted with 100 mL methanol–water solution (70:30 *v*/*v*) containing 5 g of sodium chloride to enhance extraction efficiency. The mixture was stirred at 200 rpm for 30 min at room temperature (approximately 22 °C), then filtered. 5 mL of the resulting extract was filtered, diluted with water (1:4), and purified using an immunoaffinity column (3 mL AFLASTAR^®^ IA, Romer Labs, Butzbach, Germany) specific for aflatoxins B1, B2, G1, and G2.

The methanol eluate was collected and injected into an HPLC system (Perkin Elmer 200 Series, Waltham, MA, USA) equipped with a fluorescence detector and a post-column derivatization unit (Romer Labs, Butzbach, Germany). Separation was achieved on a Tracer Excel 120 ODS-B column (5 μm, 150 × 4.6 mm, Teknokroma Analítica, Barcelona, Spain) under isocratic conditions at a flow rate of 0.7 mL/min. The column compartment was maintained at 35 °C, with excitation and emission wavelengths set at 360 nm and 440 nm, respectively. The injection volume was 40 μL.

### 2.11. Amino Acid Profile Analysis

The amino acid profile was determined following a modified version of the method by Synaridou et al. Approximately 5 g of homogenized sample was hydrolyzed with 20 mL of 4 M HCl at 95 °C for 24 h. After hydrolysis, the solution was filtered, neutralized with 15 mL of 10% potassium hydroxide (KOH) solution, and re-filtered through a 0.45 μm membrane filter.

Samples were derivatized pre-column with ortho-phthalaldehyde (OPA), used as supplied by the manufacturer without further dilution, in a 1:2 ratio using an automated sample injection system. Analysis was performed using a Vanquish UHPLC system (Thermo Fisher Scientific, Waltham, MA, USA) equipped with a fluorescence detector, set at excitation and emission wavelengths of 340 nm and 450 nm, respectively. Chromatographic separation was achieved on a Hypersil Gold C18 column (150 × 4.6 mm, 5 μm particle size) using a gradient of ultrapure water and acetonitrile at a flow rate of 0.800 mL/min. The injection volume was 1 μL, and the column temperature was maintained at 25 °C.

### 2.12. Protein Solubility Determination

Protein solubility was determined by dispersing 100 mg of protein sample in 10 mL of distilled water. The mixture was stirred at room temperature for 30 min using a magnetic stirrer to ensure complete homogenization. After agitation, the solution was centrifuged at 5000 rpm for 10 min to separate the soluble fraction from the insoluble residue. The supernatant was collected, and the protein content in the soluble fraction was measured using the Kjeldahl method as described previously. Solubility was calculated as the percentage of soluble protein relative to the initial total protein content.

### 2.13. Elemental Content Determination

For the determination of metal concentrations, samples were first dried at 105 °C and then ground into a fine powder. Approximately 0.5 g of each sample was weighed into Berzelius beakers, to which 6 mL of 65% (*w*/*w*) nitric acid (HNO_3_) and 2 mL of 30% (*w*/*w*) hydrogen peroxide (H_2_O_2_) were added. The samples were left to digest at room temperature for 16 h, followed by refluxing on a sand bath at 120 °C for 2 h.

The resulting solutions were filtered through 0.45 μm porosity filter paper into 50 mL volumetric flasks and brought to volume with ultrapure water.

Metal concentrations in the digested solutions were determined by inductively coupled plasma optical emission spectrometry (ICP-OES) using an Optima 5300 DV spectrometer (Perkin Elmer, Waltham, MA, USA). The operating parameters included a free-running generator at a frequency of 40.68 MHz, dual-view plasma observation (axial and radial), and an argon gas flow rate of 17 L/min. Calibration curves were prepared in six points over the concentration range of 0–10 mg/L using a multielement standard solution (ICP Standard IV, 1000 mg/L, Merck, Darmstadt, Germany).

### 2.14. Statistical Evaluation

All experiments were conducted in triplicate, and results are expressed as mean values ± standard deviations. Statistical analyses were performed using Minitab for Windows version 17.0 (Minitab, LLC, State College, PA, USA). A Taguchi L9 orthogonal array design was employed to investigate the influence of three independent variables—pH, temperature, and sample mass—on protein extraction content, each tested at three levels. The three independent variables evaluated were pH (9.5, 10.0, and 10.5), temperature (30, 40, and 50 °C), and sample mass (40, 50, and 60 g per 500 milliliters of distilled water), each tested at three levels. Analysis of variance (ANOVA) was applied to evaluate the statistical significance of individual factors and their contributions to the response. Additionally, a first-order linear regression model was developed using Minitab to describe the relationship between the selected process variables and protein content. The model was further assessed using diagnostic tools such as residual analysis to verify compliance with underlying statistical assumptions. Statistical significance was considered at a 95% confidence level (*p* < 0.05).

## 3. Results and Discussion

### 3.1. Nutritional Composition of Sunflower Meal

The proximate composition of the sunflower meal used in this study is presented in Table 1. The material exhibited a high dry matter content of 99.8%, indicating very low residual moisture and thus good storage stability. The protein content was 29.73%, confirming the potential of sunflower meal as a valuable alternative protein source. A high fiber content of 51.8% was observed, characteristic of sunflower meal and a factor that can influence extraction efficiency due to the presence of complex plant cell wall structures.

The lipid content was relatively low at 1.2%, reflecting the effectiveness of the cold-press oil extraction process. Ash content reached 10.87%, suggesting a significant contribution of mineral compounds. The observed standard deviations, generally around 10–12% of the measured values, are consistent with natural variability in sunflower meal composition due to seed origin, processing conditions, and post-extraction handling. Overall, the results confirm that sunflower meal represents a nutrient-dense by-product suitable for further protein extraction and valorization. The protein content of 29.73% observed in this study aligns with previously reported values in the literature, which typically range from 28–30% for sunflower meal with hulls [33]. This is consistent with other studies that have reported protein contents between 28.0% and 39.9% depending on the processing method [34]. The high fiber content (51.8%) observed in our study was notably higher than typical values reported in the literature, which range from 25% for meals with hulls to 15% for completely dehulled meals [3]. This higher fiber content suggests minimal dehulling in our starting material, which could impact the subsequent protein extraction efficiency.

The fat content (1.2%) falls within the expected range of 0.9–10.3% reported in previous studies [28], and is particularly consistent with values typically observed in solvent-extracted meals (1–2%) [35]. This low residual oil content is advantageous for protein extraction as it minimizes potential interference from lipids during the process.

### 3.2. Comparative Evaluation of Extraction Methods

The extraction protein contents obtained using different methods are presented in Table 2. Among the three evaluated techniques, CO_2_-assisted extraction demonstrated the highest protein content, achieving 44.23 ± 4.1% (dry basis). Mechanical stirring resulted in an intermediate protein content of 41.52 ± 4.2%, while ultrasound-assisted extraction produced a slightly lower protein content of 39.14 ± 3.5%.

The superior performance of the CO_2_-assisted method can be attributed to the enhanced mass transfer and cell disruption facilitated by the combined effects of pressure, agitation, and CO_2_ solubilization. The superior performance of CO_2_-assisted extraction (44.23%) compared to mechanical stirring (41.52%) and ultrasound-assisted extraction (39.14%) aligns with previous findings on the effectiveness of supercritical CO_2_ extraction in preserving protein functionality while achieving higher yields [11]. However, ultrasound-assisted extraction in our study showed lower efficiency than some previous reports, where UAE has demonstrated significant improvements in protein yield and functional properties [36]. This difference might be attributed to variations in ultrasound parameters and the high fiber content of our starting material, which could have impeded the effectiveness of cavitation.

These conditions likely promoted a more efficient release of intracellular proteins without causing significant thermal degradation, as the extraction was conducted at relatively low temperatures (20.7–22.3 °C). In contrast, mechanical stirring and ultrasound-assisted extraction, while effective, may have been limited by lower penetration into the complex fiber-rich structure of the sunflower meal matrix.

The results underline the potential of CO_2_-assisted extraction as an efficient and gentle technique for recovering proteins from plant by-products with high fiber content, supporting its application in sustainable food processing strategies. Statistical analysis of the protein content obtained from the three extraction methods was conducted using one-way ANOVA followed by Tukey’s post hoc test. The results showed no statistically significant differences among the methods (*p* > 0.05), indicating that all approaches yielded comparable protein extraction efficiency.

### 3.3. Experimental Design, Modeling, and Optimization Validation

Although CO_2_-assisted extraction achieved a slightly higher protein recovery (44.23 ± 4.1%) compared to mechanical stirring (41.52 ± 4.2%), the latter was selected for further optimization due to practical and economic considerations. CO_2_-assisted extraction requires specialized high-pressure reactors and compressors, with reported energy consumption exceeding 1.5–2 kWh per batch for agitation and CO_2_ cycling. In contrast, mechanical stirring consumes considerably less energy (<0.2 kWh per batch) and employs cost-effective, widely available equipment. Additionally, the capital investment required for CO_2_ extraction systems can be 5–10 times higher than that for mechanical stirrers, limiting scalability for small- and medium-sized food processing facilities. From a safety standpoint, CO_2_ systems also necessitate pressure handling protocols and operator training. Given the marginal protein content difference (~2.7%), mechanical stirring offers a more energy-efficient, cost-effective, and scalable alternative, aligning with sustainable production goals.

To optimize the protein extraction process using mechanical stirring, a factorial experimental design based on a Taguchi L9 orthogonal array was applied. The three independent variables investigated were pH (10.0, 10.5, 11.0), temperature (30 °C, 40 °C, 50 °C), and sample mass (40 g, 50 g, 60 g per 500 mL of distilled water), each at three levels (Table 3). This design enabled the systematic assessment of main effects and interactions using a reduced number of experimental runs (Table 3). Experimental design for protein extraction optimization (Taguchi L9 array).

**Figure 1:**
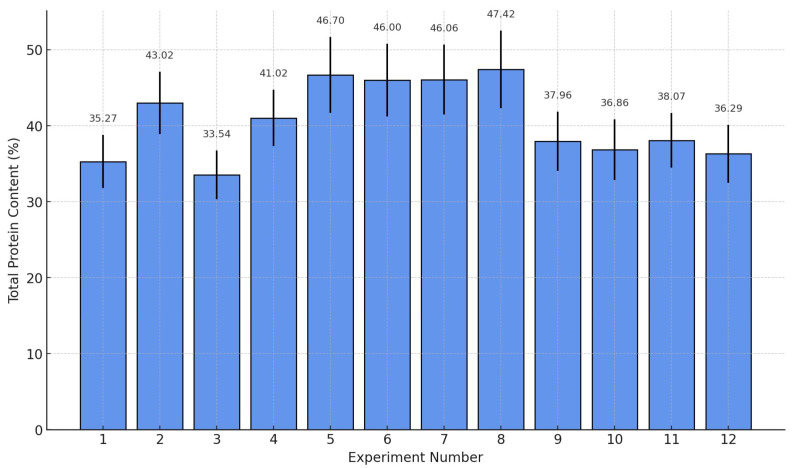
Protein content from Taguchi L9 design with experimental variability.

The experimental results (Figure 1) revealed significant variability in protein extraction efficiency, with contents ranging from 33.54% to 47.42%. Among the tested conditions, the combination of pH 11.0, temperature 50 °C, and 60 g sample mass provided the highest protein content (47.42%). Statistical analysis confirmed no significant differences between the protein contents of samples 2 and 4–8 (*p* > 0.05), supporting the model’s consistency in identifying optimal extraction conditions protein extract.

To better understand the effects of the tested parameters, a multiple linear regression model was developed based on the Taguchi L9 design data. Although signal-to-noise ratio analysis is traditionally used in Taguchi methods, it was not applied in this study. Instead, regression modeling and ANOVA were used to identify the influence and significance of individual factors. This approach was chosen to focus on maximizing mean protein yield rather than evaluating performance variability. The model aimed to quantify the effects of temperature (X2), sample mass (X3), and pH (X1) on protein content. Based on preliminary regression diagnostics and statistical significance, the variable X1 (pH) did not contribute meaningfully to the model and was therefore excluded from the final regression equation. The refined model includes temperature (X2), sample mass (X3), their interaction, and a quadratic term for temperature. The resulting equation was:

(1) $$Total\;Protein\;Content\;\left(\%\right)=53.4-2.958\cdot X2+1.145\cdot X3+0.05084\cdot {X2}^{2}-0.01821\cdot X2\cdot X3$$

The analysis of variance (ANOVA) for this model is shown in Table 4. Among the factors, sample mass had a statistically significant effect (*p* = 0.0049), accounting for approximately 69.66% of the total variance. Temperature contributed 15.05% but was not statistically significant (*p* = 0.115). pH exhibited minimal influence (*p* = 0.809) and contributed only 1.05%, justifying its exclusion from the final regression model. The model demonstrated excellent predictive capacity, with R^2^ = 97.45% and adjusted R^2^ = 96.2%.

**Figure 2:**
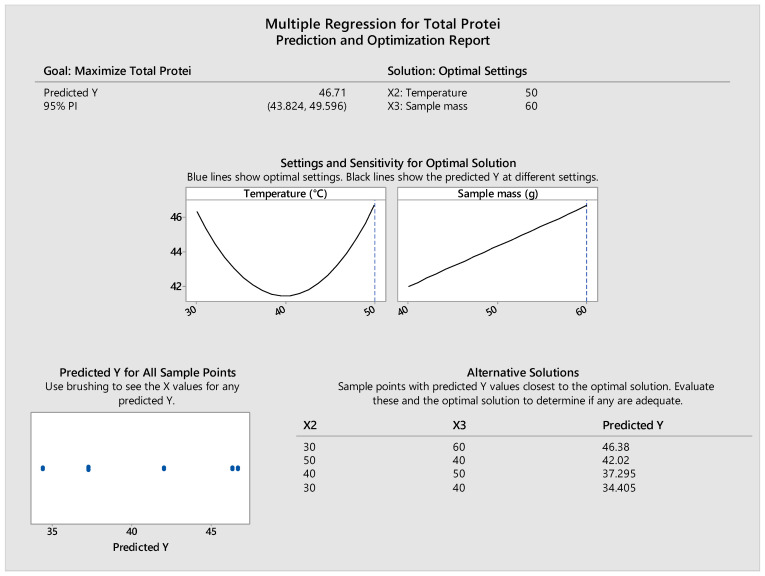
Optimization report for total protein content generated via Minitab showing predicted content, optimal settings, sensitivity analysis, and alternative solutions.

The model identified the optimal conditions for protein extraction as 50 °C and 60 g sample mass per 500 mL water, predicting a protein content of 46.71% with a 95% prediction interval of 43.82–49.60% (Figure 2).

The sensitivity analysis showed that increasing sample mass had a consistently positive effect on protein content, while temperature exhibited a quadratic response, peaking at 50 °C. Several alternative solutions with similar predicted contents were also identified, reinforcing the model’s robustness.

**Figure 3:**
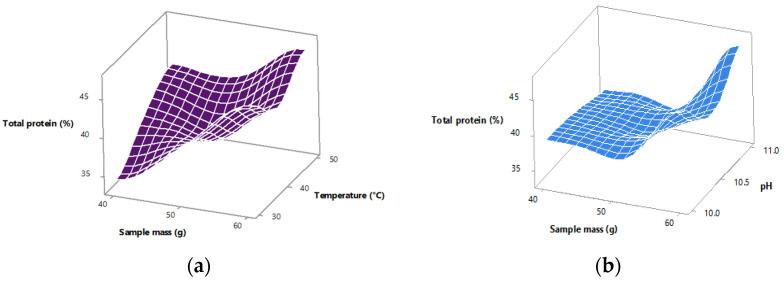
Surface plots showing the effect of: (**a**) temperature and sample mass; and (**b**) pH and sample mass on total protein content (%).

Figure 3 displays three-dimensional surface plots of protein content in relation to the studied variables. Figure 3a shows the interaction between temperature and sample mass, with pH held constant. The plot indicates that content increases with both temperature and sample mass, confirming the importance of these variables. Figure 3b presents the effect of pH and sample mass, with temperature held constant. The relatively flat slope for pH corroborates the statistical insignificance observed in the ANOVA, while sample mass again shows a strong positive trend.

To validate the optimization results, the predicted optimal conditions were tested experimentally at pH 10, temperature 30 °C, and 60 g of sunflower meal in 500 mL of distilled water. The measured protein content was 47.52%, which aligns closely with the predicted value of 46.71%, demonstrating the accuracy and applicability of the model.

These findings confirm the effectiveness of Taguchi design combined with multiple regression modeling as a reliable approach for optimizing protein extraction from agro-industrial by-products like sunflower meal, supporting circular economy goals and sustainable food production. To assess the robustness of the developed model, three additional experimental conditions were tested outside the predicted optimum. As shown in Table 5, the experimentally obtained total protein content closely matched the predicted values, with deviations ranging from –1.1% to +1.4%. These small errors confirm the model’s predictive accuracy and its applicability across a broader parameter range, not limited to the optimized conditions.

### 3.4. Characterization of Protein Extract

The proximate composition of the optimized sunflower protein concentrate obtained from sunflower meal is presented in Table 6. Compared to the original sunflower meal, significant improvements in protein concentration were observed. The extracted protein flour exhibited a protein content of 49.87 ± 5.2% (dry basis), substantially higher than the 29.73 ± 3.4% protein content recorded in the raw material. This enhancement confirms the effectiveness of the extraction and purification processes in concentrating the protein fraction. This moderate 49.87% protein concentration level may be attributed to the high initial fiber content of our starting material. The solubility (53.4%) aligns with values for mildly extracted plant proteins [37], though some studies have reported higher solubility values through enzymatic modification or pH optimization [38].

The mineral profile of our extract shows notable differences from previously reported values. The high sodium and potassium contents (867.7 and 764.7 mg/kg, respectively) are particularly noteworthy, as they exceed typical levels reported in the literature. This enrichment might be attributed to the alkaline extraction conditions used in our process.

The fat content of the extract remained low, at 1.01 ± 0.08%, comparable to that of the starting material. A notable reduction in fiber content was achieved, decreasing from 51.8 ± 5.9% in the raw meal to 13.24 ± 1.4% in the extract, indicating effective separation of insoluble matrix components during processing. The ash content was measured at 8.91 ± 0.9%, slightly lower than in the raw sunflower meal, suggesting partial removal of mineral content during extraction. The nutritional characterization highlights that the optimized extraction process effectively concentrated the protein fraction while reducing undesirable components such as fiber and ash, thus producing a higher-value ingredient suitable for human nutrition applications.

Compared to other commonly used plant protein sources, the optimized sunflower protein flour demonstrates a competitive nutritional profile. The protein content of 49.87 ± 5.2% is comparable to soy flour (typically 45–50%) and pea protein isolates (around 50–55%), while maintaining a low-fat content (1.01 ± 0.08%) [39,40,41,42,43,44]. The fiber content (13.24 ± 1.4%) further enhances its functional and nutritional value. The mineral composition is also favorable, with higher potassium and sodium concentrations than values reported for untreated sunflower meal, potentially due to the alkaline extraction process [43]. These attributes suggest that the extract is not only a sustainable alternative protein source but also a nutritionally relevant ingredient suitable for incorporation into food formulations targeting protein enrichment and dietary diversification.

In addition to the proximate composition, the solubility of the extracted sunflower protein flour was evaluated, an important functional property for potential food applications. The sunflower protein flour exhibited a solubility degree of 53.4%, which is considered satisfactory for plant-based proteins obtained through mild extraction processes. This moderate solubility indicates that the extract retains a significant fraction of water-dispersible proteins, making it suitable for incorporation into beverages, emulsions, and other functional food matrices.

The mineral composition of the extracted sunflower protein flour is presented in Table 7. The extract exhibited significant levels of essential minerals, confirming its potential to contribute not only as a protein source but also as a micronutrient-rich ingredient (Table 7). Among the elements analyzed, potassium (K) and sodium (Na) were the most abundant, with concentrations of 764.7 mg/kg and 867.7 mg/kg, respectively. Magnesium (Mg) was also present at a notable level of 219.3 mg/kg, followed by calcium (Ca) at 549.9 mg/kg. These minerals are essential for various physiological functions, including muscle contraction, electrolyte balance, and bone health. Trace elements such as iron (Fe) and copper (Cu) were detected at lower concentrations, 1.7 mg/kg and 13.0 mg/kg, respectively. Iron is critical for oxygen transport and enzymatic processes, while copper contributes to antioxidant defenses and connective tissue formation. The presence of these essential minerals enhances the nutritional value of the sunflower protein concentrate and supports its positioning as a functional food ingredient with added health benefits.

### 3.5. Amino Acid Profile

**Figure 4:**
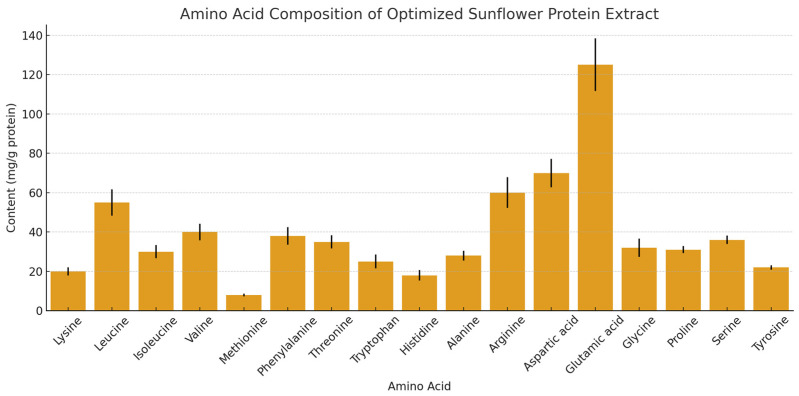
Amino acid composition of the optimized sunflower protein extract, expressed as mean ± standard deviation (mg/g protein, *n* = 3). Values were determined in triplicate using the extract obtained under optimal processing conditions.

The amino acid profile of the optimized protein concentrate is presented in Figure 4. All measurements were performed in triplicate, and results are expressed as mean values with standard deviation to reflect analytical reproducibility. Both essential and non-essential amino acids were identified, confirming the nutritional potential of the extract for human consumption.

Among the non-essential amino acids, glutamic acid and aspartic acid were predominant, with glutamic acid showing the highest concentration. These amino acids are known for contributing to the flavor profile and enhancing the functional properties of plant-based proteins. A high level of arginine was also observed, which is notable given its role in cardiovascular health and nitrogen metabolism. Regarding essential amino acids, lysine and methionine were present but at comparatively lower concentrations than other amino acids. This observation aligns with previous findings for sunflower proteins and highlights the typical limitation in sulfur-containing amino acids common among plant proteins.

Nevertheless, the presence of all essential amino acids, albeit in varying amounts, supports the potential of sunflower protein extracts to contribute to a balanced dietary amino acid intake, especially when combined with complementary protein sources. The amino acid profile of the extract reinforces its applicability as a functional ingredient in food formulations aimed at improving protein quality and diversity. The predominance of glutamic and aspartic acids in our extract is consistent with previous characterizations of sunflower proteins [44], where these amino acids typically constitute the major fraction of the protein. The relatively lower concentrations of lysine and methionine observed in our study are characteristic of sunflower proteins, as reported in multiple studies [45]. This amino acid distribution confirms the typical pattern of sunflower proteins, where globulins (particularly helianthinin) constitute the major protein fraction in a ratio of approximately 2:1 to albumins [46].

To further evaluate the nutritional quality of the extracted protein, its essential amino acid composition was compared with the WHO/FAO/UNU reference amino acid scoring patterns for adult human nutrition [47]. The extract showed high levels of leucine and valine, which are branched-chain amino acids important for muscle metabolism, as well as appreciable amounts of lysine, which is often limiting in plant-based proteins. Although the sulfur-containing amino acids methionine and cysteine were present at lower levels, the overall profile indicates that the extract can serve as a valuable complementary protein source in plant-based diets or blended formulations.

### 3.6. Contaminant Evaluation

The safety of the extracted sunflower protein flour was evaluated by analyzing potential chemical contaminants, specifically polycyclic aromatic hydrocarbons (PAHs) and aflatoxins. The concentrations of 15 PAHs were assessed. Naphthalene, phenanthrene, fluorene, fluoranthene, and pyrene were detected at trace levels, with concentrations ranging from 0.0021 to 0.0038 µg/kg. The remaining PAHs were below the limit of quantification (LQ ≥ 0.0001 µg/kg). The total PAH content was calculated at 0.0115 µg/kg, significantly below the thresholds considered hazardous for food products. These results indicate a minimal risk of contamination and confirm the suitability of the extract for human consumption from a chemical safety perspective. The trace levels of PAHs detected in our extract (0.0021 to 0.0038 µg/kg) are well below the maximum levels established by regulatory bodies [41]. Similarly, the non-detectable levels of aflatoxins (below 0.04 µg/kg for B1 and G1, and below 0.15 µg/kg for B2 and G2) are significantly lower than the EU regulatory limits of 2.0 µg/kg for aflatoxin B1 and 4.0 µg/kg for total aflatoxins [48]. These results demonstrate excellent safety profiles compared to reported contamination levels in similar plant-based protein products [49,50], indicating effective quality control during processing and storage.

The contents of aflatoxins B1, B2, G1, and G2 were below their respective limits of quantification (LQ1 ≥ 0.04 µg/kg for B1 and G1; LQ2 ≥ 0.15 µg/kg for B2 and G2). No detectable levels of aflatoxins were observed in the analyzed samples, highlighting good raw material quality and appropriate storage and handling conditions during processing [51].

The extracted sunflower protein flour exhibited negligible contamination, fulfilling safety requirements for use in human nutrition.

These results reinforce the feasibility of integrating sunflower protein extracts into human diets as part of a broader strategy to utilize underexploited plant resources. Future research should focus on improving the functional properties of sunflower proteins—such as solubility and emulsifying capacity—and evaluating their performance in complex food matrices. Furthermore, sensory evaluation and clinical nutritional studies would support market acceptance and regulatory approval of such novel protein ingredients.

Compared to previous studies on protein extraction from sunflower meal, the optimized conditions identified in this work include a sample mass of 60 g per 500 milliliters, a temperature of 50 degrees Celsius, and a pH of 10.5. This combination resulted in improved protein yield and functional characteristics. Earlier research mainly focused on maximizing extraction efficiency using single variable approaches or less efficient experimental designs. In contrast, our use of a Taguchi orthogonal array enabled a structured evaluation of the most influential factors with a reduced number of experiments. The resulting protein concentrate was thoroughly characterized in terms of amino acid composition, mineral content, and solubility, offering valuable insight into its potential use in food applications. These findings contribute new knowledge by proposing a reproducible and scalable method that supports both the nutritional and functional valorization of sunflower meal as a sustainable protein source.

## 4. Conclusions

This study demonstrated that sunflower meal, a widely available agro-industrial by-product, can be effectively valorized as a protein source through optimized alkaline extraction. Using a Taguchi L9 design and regression modeling, we identified key process parameters—particularly sample mass and temperature—that significantly influenced protein content. The optimized method achieved a protein content of 47.87%, closely aligning with model predictions and confirming the method’s reliability. Beyond confirming the feasibility of protein recovery, these findings contribute to the advancement of sustainable food systems by supporting the circular use of agricultural waste. The extracted protein flour exhibited favorable nutritional characteristics, suggesting its potential application in functional foods and meat alternatives. Future research should focus on expanding the characterization of the extracted proteins beyond yield and solubility. In particular, assessing protein purity using SDS-PAGE or advanced Kjeldahl-based fractionation and evaluating functional properties such as emulsification capacity, gelation, and foaming ability are necessary to determine suitability for complex food systems. Additionally, testing the incorporation of sunflower protein flour into model food matrices, alongside sensory and shelf-life analyses, would provide valuable data to support food industry applications. These efforts will strengthen the scientific basis for the use of sunflower meal as a sustainable, functional protein source for human nutrition.

## Figures and Tables

**Table 1:** Nutritional composition of sunflower meal (mean ± standard deviation, *n* = 3).

Parameter	Unit	Value
Dry matter	%	99.8 ± 0.5
Fat	%	1.2 ± 0.1
Protein	%	29.73 ± 3.4
Fiber	%	51.8 ± 5.9
Ash	%	10.87 ± 1.3

**Table 2:** Protein content after extraction (mean ± standard deviation, *n* = 3).

Extraction Method	Total Protein Content (% Dry Basis) *
Mechanical stirring	41.52 ± 4.2
Ultrasound-assisted extraction	39.14 ± 3.5
CO_2_-assisted extraction	44.23 ± 4.1

* No statistically significant differences were observed between extraction methods (*p* > 0.05, one-way ANOVA with Tukey’s test.

**Table 3:** Experimental design for protein extraction optimization (Taguchi L9 array).

Experiment	pH	Temperature (°C)	Sample Mass (g/500 mL)
1	10	30	40
2	10	50	40
3	11	30	40
4	11	50	40
5	10	30	60
6	10	50	60
7	11	30	60
8	11	50	60
9	10.5	40	50
10	10.5	40	50
11	10	40	50
12	11	40	50

**Table 4:** ANOVA Table for Protein Content.

Factor	Sum of Squares	Degrees of Freedom	F-Value	*p*-Value	Contribution (%)
pH	2.29	2	0.22	0.809	1.05
Temperature	32.98	2	3.17	0.115	15.05
Sample Mass	152.62	2	14.68	0.0049	69.66
Residual	31.20	6	—	—	14.24

**Table 5:** Validation of the regression model at additional experimental conditions outside the predicted optimum.

Condition Tested (pH, Temp, Mass)	TPC Predicted (%)	TPC Experimental (%)	Error
10.5, 35 °C, 50 g	42.5	43.1	+1.4
11.0, 40 °C, 60 g	45.8	44.7	–1.1
10.0, 50 °C, 60 g	46.5	46.0	–0.5

**Table 6:** Proximate composition of the extracted sunflower protein flour (mean ± standard deviation, *n* = 3).

Parameter	Unit	Value
Fat	%	1.01 ± 0.08
Protein	%	49.87 ± 5.2
Fiber	%	13.24 ± 1.4
Ash	%	8.91 ± 0.9

**Table 7:** Mineral composition of the optimized extracted sunflower protein flour.

Element	Concentration (mg/kg)
Iron (Fe)	1.7 ± 0.2
Copper (Cu)	13.0 ± 1.1
Calcium (Ca)	549.9 ± 12.5
Sodium (Na)	867.7 ± 14.2
Potassium (K)	764.7 ± 10.3
Magnesium (Mg)	219.3 ± 7.6

## Data Availability

The data presented in this study are available upon request from the corresponding author. The data are not publicly available due to privacy restrictions.
